# 

**Published:** 1914-06

**Authors:** 


					﻿gshj wdyki k iqikmkq q
VoLXXlX}
‘JUNE,>914
NO 12
"Red Back"
TEXasIM EllCAU W^naeg
csayug jully 1885
KYXXX MMu;'A AT A LS H X, ': blfo,
ffWOrTWi iitM
rlil?rr<£’) BY T.K?-: YOIY BOErYAVANY-.iOK.ES C'd. -■
Sttb’serintWu.- S1.CM a Yeasx■ 1 scksajcis
Eatered at the Pfist^tiice 4t A<u;»jii,'	.Uk
»
■
il
■
B
ps®4''
z
■
■
8
H
■
■
				

